# New Closure Method Using Loop and Open–Close Clips after Endoscopic Submucosal Dissection of Stomach and Colon Lesions

**DOI:** 10.3390/jcm10153260

**Published:** 2021-07-23

**Authors:** Akira Yoshida, Hiroki Kurumi, Yuichiro Ikebuchi, Koichiro Kawaguchi, Kazuo Yashima, Yu Kamitani, Sho Yasui, Yusuke Nakada, Tsutomu Kanda, Tomoaki Takata, Hajime Isomoto

**Affiliations:** Division of Gastroenterology and Nephrology, Faculty of Medicine, Tottori University, Tottori 683-8504, Japan; kurumi_1022_1107@yahoo.co.jp (H.K.); ikebu@tottori-u.ac.jp (Y.I.); koichiro@med.tottori-u.ac.jp (K.K.); yashima@med.tottori-u.ac.jp (K.Y.); yukamitani@aol.com (Y.K.); n9k_htke@yahoo.co.jp (S.Y.); xeph@hotmail.co.jp (Y.N.); tsutomu.kanda.s@gmail.com (T.K.); t-takata@tottori-u.ac.jp (T.T.); isomoto@tottori-u.ac.jp (H.I.)

**Keywords:** clip closure, endoscopic submucosal dissection (ESD), stomach cancer, colon cancer

## Abstract

Endoscopic submucosal dissection (ESD) and *en bloc* resection of stomach and colon tumors have become common. However, mucosal defects resulting from ESD may cause delayed bleeding and perforation. To prevent adverse events, we developed a new clip closure technique, namely, the loop and open–close clip closure method (LOCCM), and aimed to examine its efficacy after ESD for stomach and colon tumors. The LOCCM uses loop and open–close clips. Here, the open–close clip was used to grasp the loop to bring it to the edge of the post-ESD mucosal defect. Another clip with a loop was then inserted into the opposite edge and clipped to the contralateral mucosa to pull both edges together. Once apposed, additional clips facilitated complete closure. The LOCCM was performed in 19 patients after ESD at Tottori University between October 2020 and March 2021. The outcomes retrospectively analyzed were the LOCCM success and adverse event rates. The complete closure rate using LOCCM was 89.5% and none of the patients had post-ESD bleeding or perforation. The results show that LOCCM is an effective and safe closure technique for mucosal defects after stomach and colon ESD to prevent bleeding and perforation.

## 1. Introduction

Endoscopic submucosal dissection (ESD) is becoming the standard of care for the resection of early gastric cancer with a confirmed diagnosis [1,2,3,4,5]. Colorectal ESD treatment has become widespread due to recognition of its efficacy and safety [5,6,7]. However, compared to other treatments such as endoscopic mucosal resection (EMR), ESD is associated with a higher risk of postoperative side effects, such as bleeding and perforation [5,8,9], due to the large size of the mucosal defects. In addition, the use of antithrombotic drugs, which has been increasing in recent years, is also a known contributory factor. In cases of delayed perforation after stomach ESD, emergency surgery is required [10]. Treatment of mucosal defects after ESD is crucial. Polyglycolic acid sheet shielding is one method for mucosal defect treatment [11]. However, closure of the mucosal defect is considered the most effective method. Clip closure accelerates the healing of mucosal defects [12] and prevents adverse events, such as delayed bleeding and perforation, after EMR and ESD [13,14]. Closure of mucosal defects after stomach and colorectal ESD is challenging; however, several endoscopic closure techniques have been reported to be useful. Currently, there is no standard closure method for mucosal defects. Clip-on-clip closure, hand-suturing, string clip suturing, mucosa–submucosa clip closure, hold-and-drag closure, closure with a detachable snare and clips, over-the-scope clip, and through-the-scope clip are some methods that have been reported, with success rates of 62–97% [15,16,17,18,19,20,21]. The primary problem with closure is how to best bring the bilateral edges of the mucosal defect together. In this study, we report a new, simple closure method using loop and open–close clips that make it possible to easily appose contralateral mucosa. We retrospectively evaluated the feasibility of the loop and open–close clip closure method (LOCCM) for mucosal defects after ESD.

## 2. Materials and Methods

### 2.1. Study Population

This study included patients with gastric or colon neoplasms resected using ESD at Tottori University Advanced Endoscopic Center between October 2020 and March 2021. Gastric ESD and colon ESD were performed using the GIF-H290T and PCF-H290TI scopes (Olympus Corp., Tokyo, Japan), respectively. Closure of the mucosal defect after ESD was performed by an experienced endoscopist. The exclusion criteria were as follows: (1) gastric tumor extension to the cardia or pyloric ring; (2) colon tumor extension to Bauhin’s valve and anal canal; (3) circumferential lesions of the stomach and colon; and (4) lack of patient consent. This study was approved by the Ethics Committee of Tottori University (approval number: 1508A024).

### 2.2. The Loop and Open–Close Clip Closure Method

**Figure 1:**
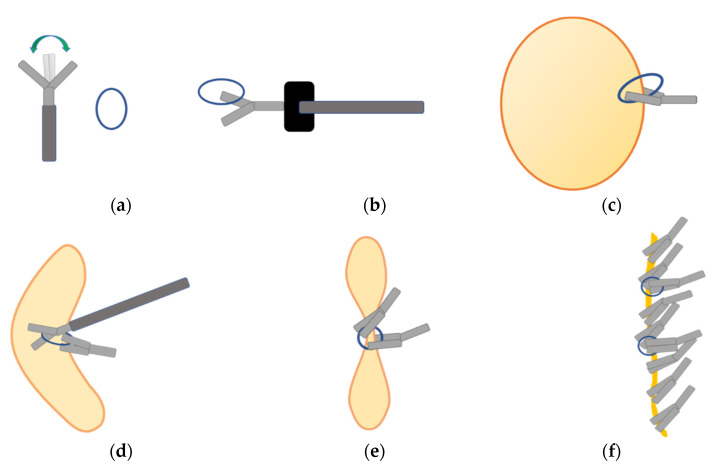
(**a**) An open–close clip (SureClip) and loop were prepared. (**b**) After removal of the forceps plug from the endoscope, the SureClip was passed through the forceps plug and then opened to grasp the loop. (**c**) The forceps plug was reattached to the endoscope, and the clip was advanced with the loop to bring it to the edge of the post-ESD mucosal defect. The first clip with a loop grasped proximal to the center of the mucosal defect. (**d**) The next clip was used to drag the loop and grasp the distal side of the mucosal defect. (**e**) After placing the second clip, we can reach the closer bilateral mucosa. (**f**) After the addition of the second clip, both edges of the mucosal defect were adjacent, allowing the addition of SureClips or conventional clips to close the mucosal defect completely. If the bilateral edges of the defect were still at considerable distance, the same process of using a loop and clip was carried out to bring the edges of the defect closer. The procedure was completed once the mucosal defects were completely closed.

**Figure 2:**
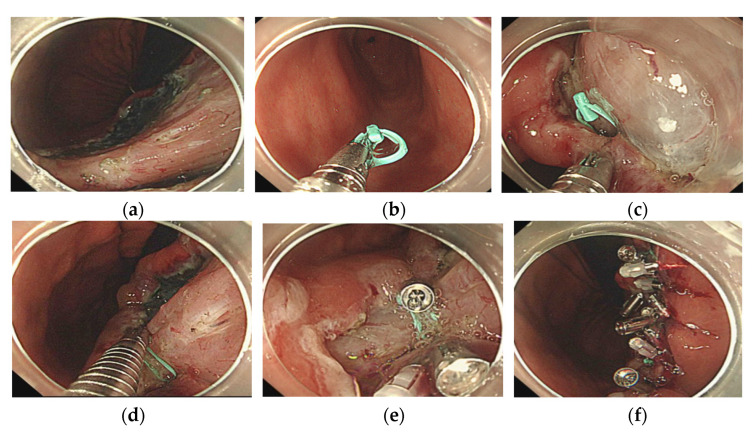
(**a**) A gastric angle mucosal defect occurred subsequent to endoscopic submucosal dissection. (**b**) SureClip with the loop is delivered to the mucosal defect. (**c**) SureClip with the loop is clipped to the anal side of the mucosal defect. (**d**) Another clip is placed and used to drag the loop to the oral side of the mucosal defect. (**e**) Both edges of the mucosal defect are observed to be adjacent. (**f**) Clips are added for complete and adequate closure of the mucosal defect.

**Figure 3:**
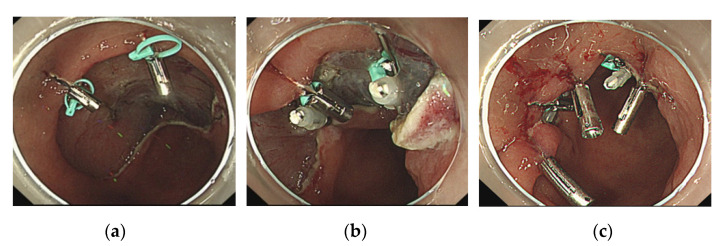
(**a**) A mucosal defect of the anterior wall of the gastric antrum occurred after endoscopic submucosal dissection. Two SureClips with the loops are delivered and clipped to the edges of the mucosal defect. (**b**) Two other clips are placed on the opposite side and used to drag the loop and appose the edges of the mucosal defect. (**c**) Clips are added for complete and adequate closure of the mucosal defect.

**Figure 4:**
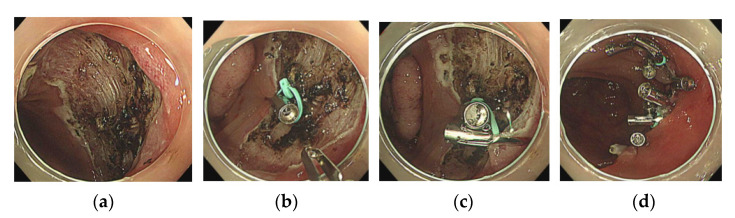
(**a**) A mid-transverse colon mucosal defect occurred subsequent to endoscopic submucosal dissection. (**b**) SureClip with the loop is delivered to the edge of the mucosal defect. (**c**) Another clip is placed on the opposite side of the mucosal defect and used to drag the loop. (**d**) Clips are added for complete and adequate closure of the mucosal defect.

Closure using LOCCM was performed on the mucosal defect after ESD of the stomach and colorectal lesions. The new closure was performed in ten stomach and nine colon lesion cases. This closure method uses a loop, which is part of the S-O clip (Zeon Medical, Toyama, Japan) [22], open–close SureClip clips (11 mm, 16 mm) (Microtech, MI, USA) [23], and conventional HX-610-090S/HX-610-090/HX-610-090L/HX-610-135L clips (Olympus, Tokyo, Japan). All LOCCMs were performed with a single channel using the same scope as ESD (GIF-H290T, PCF-H290TI; Olympus, Tokyo, Japan). LOCCM was performed as follows (Figure 1, Figure 2, Figure 3 and Figure 4):

1. An open–close clip (SureClip) and loop were prepared;
2. After removal of the forceps plug from the endoscope, the SureClip was passed through the forceps plug and then opened to grasp the loop;
3. The forceps plug was reattached to the endoscope, and the clip was advanced with the loop to bring it to the edge of the post-ESD mucosal defect;
4. The first clip with a loop was used to grasp the center of the mucosal defect;
5. The next clip was used to drag the loop and grasp the distal side of the mucosal defect.

After the addition of the second clip, both edges of the mucosal defect were adjacent, allowing the addition of SureClips or conventional clips to close the mucosal defect completely. If the edges of the defect were still at considerable distance, the same process of using a loop and clip was performed to bring the edges of the defect closer. The procedure was completed once the mucosal defects were completely closed.

### 2.3. Evaluation of Procedural Outcomes

The successful closure rate, lesion size, lesion location, number of loops used, number of clips used, anticoagulant and antiplatelet drug use, LOCCM procedure time, and the post-ESD bleeding and perforation were evaluated as procedural outcomes. The ESD procedure time was defined as the time from the initial incision to the completion of the lesion dissection. The closure time was defined as the time from the first clipping of the LOCCM to the final clipping. Delayed bleeding was defined as the requirement for emergency endoscopic hemostasis or a hemoglobin level decrease of 2 mg/dL. Delayed perforation was defined as the onset of sudden abdominal pain and free air on CT in a patient without perforation during ESD and after resection. The observation period for complications was two months, during which the mucosal defect usually closed after ESD. The technical success rate was defined as the disappearance of the submucosal layer. Complete closure was defined as the complete disappearance of the submucosal defects. Semi-complete closure was defined as a submucosal defect that is less than 10% its original size.

### 2.4. Statistical Analysis

Each continuous variable was expressed as mean ± standard deviation. Differences between the two groups were detected using Student’s *t*-test or Welch’s *t*-test for continuous data. Statistical significance was set at *p* < 0.05. All statistical analyses were performed using the statistical software EZR version 1.54 (Saitama Medical Center, Jichi Medical University, Saitama, Japan) [24].

## 3. Results

LOCCM was applied to 19 cases (stomach, 10/19; colon, 9/19) of postoperative mucosal defects after ESD resection at Tottori University between October 2020 and March 2021. All 19 cases were resected *en bloc* by ESD without any adverse events. Pathological evaluations were subsequently performed. One patient had a remnant stomach because of a previous distal gastrectomy.

Antithrombotic agents (ATAs) were administered in 32% of the patients (6/19; stomach, 5/10; colon, 1/9). Specifically, the ATAs administered were aspirin (*n* = 1), warfarin and aspirin (*n* = 1), dabigatran, rivaroxaban, and aspirin (*n* = 1), and edoxaban (*n* = 2). ATAs were discontinued on the day of the procedure, except for the aspirin-only patient.

The pathological diagnosis was adenocarcinoma in 63% of the patients (12/19; stomach, 9/10; colon, 3/9) and adenoma in 37% (7/19; stomach, 1/10; colon 6/9) (Table 1). Invasion depth was intramucosal in 95% of the patients (18/19; stomach, 10/10; colon, 8/9), with only one colon case showing submucosal invasion. Pathological examination revealed that all tumors had been completely removed.

The complete closure rate was 89% (17/19; stomach, 8/10; colon, 9/9), while semi-complete closure was observed in 11% (2/19; stomach, 2/10; colon, 0/9) (Table 2).

The mean resected specimen size was 40.6 ± 7.1 mm (stomach, 40.4 ± 5.9; colon, 40.9 ± 8.2), while the mean tumor size was 25.2 ± 9.4 mm (stomach, 22.1 ± 8.7; colon 28.8 ± 8.8). Resection time was observed to be 89.0 ± 50.9 min (stomach, 99.7 ± 48.5; colon, 71.2 ± 47.4), while the LOCCM procedural time was 21.3 ± 10.0 min (stomach, 24.9 ± 10.9; colon, 18.7 ± 8.0). Around 9 ± 2.5 clips (stomach, 9.8 ± 2.3; colon, 8.2 ± 1.7) were used for each LOCCM procedure. The post-ESD hospitalization period was 4.3 ± 1.1 days (stomach, 4.9 ± 1.1; colon, 3.8 ± 0.8). None of the patients developed delayed bleeding or perforation.

## 4. Discussion

In this study, complete closure of the mucosal defect was achieved in 100% (9/9) of colon cases and 80% (8/10) of stomach cases. Semi-complete closure was achieved in 20% (2/10) of stomach lesions. Specifically, one case was in the lesser curvature of the gastric body, while the other was located in the posterior wall of the antrum. The two common factors observed in the two cases were poor operability of the scope and outward tension applied to the muscular layer of the stomach. However, even in the two cases of semi-completely closed stomach, the exposure of the submucosa was reduced by more than 90%, which may have prevented postoperative hemorrhage and perforation by avoiding the effects of acid and bile. The sizes of the mucosal defects in the colon were not significantly different from those in the stomach, but the closing time tended to be shorter (*p* = 0.15) and the number of clips used was fewer in the colon (*p* = 0.078), although no statistically significant difference was observed. This may be due to the thin and soft nature of the mucosal and muscular layers of the colon compared to the stomach wall, resulting in mucosal defect edges that were easier to close. A meta-analysis reported that the risk of delayed bleeding after gastric ESD was 4.1% in patients not taking ATAs and 23.4% in patients who continued to take ATAs perioperatively [25]. The delayed bleeding rate after colorectal ESD was 17.2% in all patients who underwent ATA, which was found to be higher in direct oral anticoagulant (DOAC) users than in warfarin users (23.3% vs. 11.4%) [26]. In this study, ATAs were administered to 32% (6/19) of patients, 50% (3/6) of which received DOACs. However, none of the patients experienced delayed bleeding, suggesting the efficacy of LOCCM.

This study has several limitations. This was a retrospective, single-arm study with a small number of patients. Only one experienced endoscopist was involved as a surgeon in this study. Cases involving gastric tumor extension to the cardia or pyloric ring, circumferential lesions of the stomach, and colon tumors were excluded due to the high risk of stenosis. Cases involving colon tumor extension to Bauhin’s valve and anal canal were excluded because they are anatomically difficult to close and the adverse treatment effects may greatly affect daily life. Unfortunately, rectum lesions were not included in this study and would need further investigations. The preparation of an appropriate loop was necessary. We used a 4 mm loop made of polyamide elastomer that was part of the S-O clip [22]. The rationale for removing the forceps plug before grasping the loop was to prevent the loop from being cut inside the forceps plug. The SureClip is able to achieve precise opening and closing at any point, has good rotatability, and the base of the clip can move flexibly, making it ideal for clipping any area. This makes a large contribution to the performance of the LOCCM [23]. The loop is brought to the edge of the mucosal defect, where it is opened and clipped in the optimal position. When the clip is open, the loop may be dropped into the lumen, but it can be easily retrieved. In addition, it is possible to adjust the position of the mucosal defect by grasping and pulling on the mucosa to confirm the appropriate position for closure.

For our technique, we used 4 mm ready-made loops, which are simple to use for LOCCM. However, we believe that similar silk threads can also be used as a substitute. The greatest advantage of this new technique is the ability to use more loops and open–close clips to bring the edges of the mucosal defect as close together as possible for larger post-ESD lesions, which facilitates complete closure.

## 5. Conclusions

In this study, we developed the LOCCM and found it to be a feasible technique for closing mucosal defects after stomach and colon ESD. Furthermore, it is potentially effective in decreasing the risk of post-ESD bleeding in patients at high risk due to ATA administration. Further studies are required to confirm the efficacy of the LOCCM.

## Figures and Tables

**Table 1:** Baseline characteristics of patients who underwent loop and open–close clip closure (*n* = 19).

Patient Characteristics		Value
**Patient information**		
Age (years)		71.4 ± 6.5
Stomach		72.9 ± 6.7
Colon		69.7 ± 5.9
Sex		15/19 (78.9)
Stomach Male		7/10 (70.0)
Colon Male		8/9 (88.9)
Antiplatelet therapy		3 (15.8)
Anticoagulant therapy		5 (26.3)
Antiplatelet and/or coagulant therapy		6 (31.6)
Antiplatelet and coagulant therapy		3 (15.8)
**Lesion characteristics**		
Stomach location		10 (52.6)
Lower third		2
Middle third		6
Upper third		1
Remnant		1
Stomach circumference		
Anterior wall		1
Posterior wall		3
Lesser curvature		5
Greater curvature		1
Colon location		9 (47.3)
Cecum		2
Ascending colon		3
Transverse colon		3 (hepatic flexure: 2)
Descending colon		0
Sigmoid colon		1
Rectum		0
**ESD factors**		
Procedural time (min ± SD)	Stomach	99.7 ± 48.5
	Colon	71.2 ± 47.4
En bloc resection		19 (100)
R0 resection		19 (100)
Perforation		0 (0)
**Lesion size (mm)**		
Stomach/colon resection size		40.6 ± 7.1
Stomach/colon tumor size		25.2 ± 9.4
Stomach resection size		40.4 ± 5.9
Colon resection size		40.9 ± 8.2
Stomach tumor size		22.1 ± 8.7
Colon tumor size		28.8 ± 8.8

Values are presented as number (percentage) or mean ± standard deviation. Abbreviations: LOCCM, loop and open–close clip method.

**Table 2:** Treatment outcomes of the loop and open–close clip closure method (*n* = 19).

Outcomes	Location	Value
Complete closure	Stomach and colon	17 (89)
	Stomach	8 (80)
	Colon	9 (100)
Semi-complete closure	Stomach	2 (10)
	Colon	0 (0)
Number of loops (*n* ± SD)	Stomach	2.6 ± 0.7
	Colon	1.4 ± 0.7
Number of clips used (*n* ± SD)	Stomach	9.8 ± 2.3
	Colon	8.2 ± 1.7
LOCCM time (min ± SD)	Stomach	24.9 ± 10.9
	Colon	18.7 ± 8.0
Lesion pathology		
Adenocarcinoma	Stomach	9 (90)
	Colon	3 (33)
Adenoma with high-grade dysplasia	Stomach	1 (10)
	Colon	6 (67)
Delayed bleeding		0 (0)
Delayed perforation		0 (0)
Discharge after ESD (days ± SD)	Stomach	4.8 ± 1.1
	Colon	3.8 ± 0.8

Values are presented as number (percentage) or mean ± standard deviation. Semi-complete closure was defined as submucosal defects less than 10% its original size. Abbreviations: LOCCM, loop and open–close clip method; ESD, endoscopic submucosal dissection.

## Data Availability

The data presented in this study are available on reasonable request from the corresponding author.
